# Mixing Plate-Like and Rod-Like Molecules With Solvent: A Test of Flory-Huggins Lattice Statistics

**DOI:** 10.6028/jres.100.013

**Published:** 1995

**Authors:** Edmund A. Di Marzio, Arthur J.-M. Yang, Sharon C. Glotzer

**Affiliations:** National Institute of Standards and Technology, Gaithersburg, MD 20899-0001

**Keywords:** discotic phase, Flory-Huggins, layered silicates, liquid crystals, plate-rod transition, rod-rod transition

## Abstract

Boehm and Martire have shown that the Flory-Huggins (FH) lattice model applied to mixtures of squares and rigid rods in solvent on a two dimensional lattice gives different results depending on whether rods or squares are placed first onto the lattice. This correct derivation places the validity of the FH model itself into question since the final result should be independent of the order of placement. An analysis of the FH model in terms of Poisson statistics suggests an alternative formula for the probability of successfully placing a rectangle into an area partially filled with other rectangles, which when incorporated into the FH counting procedure gives the exact thermodynamic result for the tiling of squares (i.e., no solvent and no rods). An attempt to solve the order of placement problem is made by solving the problem of one square plus any number of rods and then generalizing the statistics so that they are consistent with this result. Equations are given for squares plus rods plus solvent in both two and three dimensions. For plates plus solvent in three dimensions a purely entropy driven phase transition between an anisotropic layered phase and an isotropic phase is obtained. This transition is analogous to the isotropic to nematic liquid crystal phase transition in rigid rods. Our equations, when augmented by energy considerations, are useful for calculating the equilibrium properties of discotic systems, polymer-layered silicate composites, and the adsorption of plate like molecules onto surfaces.

## 1. Introduction

Commercial blends of polymer and layered silicates exist and have properties that are enhanced compared to those of pure polymer [1]. The economic potential of blends of polymers with inexpensive layered silicates (clays) requires us to attempt to understand the thermodynamic properties of such materials. These materials can be modeled as mixtures of plate-like molecules of nanometer thickness with flexible polymers. The discotic phase also consists of plate-like or disk-like molecules [2]. Boehm and Martire have modeled the adsorption of square plate-like molecules onto a plane surface as a mixture of plates (corresponding to molecules adsorbed face-on), rods (corresponding to edge on adsorption), and solvent on a two-dimensional lattice [3]. These three examples each require that we understand how plate-like molecules pack together in both two and three dimensions. More generally we need to know the packing statistics for mixtures of rigid plates, rods, semiflexible molecules, and solvent.

A generalization of the Flory-Huggins proceedure which can treat such systems is needed. The extension of the Flory-Huggins lattice model, which originally treated flexible molecules plus solvent, to include rigid rods and semiflexible molecules has been rather successful. First, Flory adapted his version of lattice statistics, which uses the volume fraction rather than the site fraction, to treat solutions of rigid rods of any concentration [4]. This verified the basic insight of Onsager [5] that rigid rods cannot pack at random except at very low densities (according to Flory the interference becomes appreciable at a volume fraction, *V_x_*, given by *xV_x_* = 8, *x* being the rod length). Additionally Flory’s statistics were a distinct improvement over the virial expansion approach of Onsager [5] in that they were applicable to the whole concentration range rather than only in the dilute solution range. The limitations inherent in the use of the volume fraction (for the expectation that an adjacent site is empty) were removed in a lattice treatment by Di Marzio [6]. Monte Carlo calculations have shown this expression to be accurate to within several percent over a wide range of parameters [7]. Recently Freed has given an exact expansion technique which can in principle give us results to any level of accuracy [8].

Shih and Alben [9], Herzfeld [10], and Boehm and Martire [3] have extended the statistics to plate-like objects (Shih and Alben), to arbitrary rectangular parallelepipeds (Herzfeld), and to mixtures of squares and rods on a two-dimensional lattice (Boehm and Martire). Boehm and Martire have observed that the end result for the configurational entropy of a mixture of rods and squares depends on whether the rods or squares are placed first onto the lattice in the FH counting proceedure. The answer, of course, should be independent of the order of placement. Thus, the calculation of Boehm and Martire, which was definitely done correctly, throws doubt on the validity of the Flory-Huggins procedure itself for plate-like molecules. One has always known that the FH calculation is an approximation, but the results are sufficiently divergent in this case that a reexamination of the method is demanded. Another reason for this paper is that even within the FH approximation, the results for mixtures of rods and squares in three dimensions is a useful addition to the literature.

Here we reexamine the problem of packing squares, rods and solvent on a lattice in both two and three dimensions. We begin by discussing (Sec. 2.1) the exact tiling results for the covering of a two-dimensional lattice by squares and a three-dimensional lattice by cubes. These results provide benchmarks for our final estimates. These results should be incorporated into our general formulas. A complete listing of problems for which exact results exist is small: (1) Monomers on any lattice of any dimension. (2) Space filling dimers on a two-dimensional square lattice [11]. (3) Rigid rods aligned parallel to one another, plus solvent on a hypercubic lattice of any dimension [6]. (4) The one-dimensional (*d* = 1) and *d* = ∞ cases [6]. (5) The tiling results (this paper). (6) The limit of extremely dilute solution. All of our results incorporate 1, 3 and 4: some of our results additionally incorporate 5 and 6.

Secondly, we show (Sec. 2.2) how the FH procedure can be viewed as an exercise in Poisson statistics. Poisson statistics are then used to calculate the probabilities of empty lattice sites in a field of arbitrarily distributed rigid rods. Specifically, we calculate the probability that *x* contiguous segments lying along a straight line in an arbritrary direction are empty. The procedure is then extended to square plates. In Sec. 2.3 we use these results to solve in 2-d the problem of calculating the entropy of a collection of rigid rods and solvent, and of plates and solvent. We then treat a mixture of rods, plates and solvent. Three kinds of results are obtained: (1) A result which reduces to the exact result for tiling when there are no rods or solvent, and which reduces to the rigid rod result when there are no squares; (2) The Boehm-Martire result, and (3) A modification of the Boehm-Martire result which is independent of the order of placement when many rods plus one square is placed onto the lattice. In addition, we show how to incorporate the high dilution limit exactly. In Sec. 2.4 the entropy of mixtures of squares plus rods plus solvent on a cubic lattice is obtained. It is shown that squares plus solvent undergo an entropy driven phase transition between an isotropic phase and a layered phase which is very similar to the entropy driven isotropic to nematic phase transition in rigid rod systems. For completeness, in section 2.5 the formula for the entropy of cubes in 3-d is displayed. In Sec. 3 we discuss our results.

## 2. Theory

For concreteness we discuss the problem in the context of the problem of packing squares and rods (and solvent) on a 2-d square lattice. The extension to the d-dimensional case, to rectangles, to the flexible polymer case and to the continuum case will be seen to present no additional problems of principle. Consider *M* squares occupying *r*^2^ sites each, *N*_0_ monomers, *N*_1_ rods of length *x*, and *N*_2_ rods of length *x* lying in orthogonal directions 1 and 2, respectively. The total number of lattice sites is *N* = *N*_0_ + *r*^2^*M* + *x*(*N*_1_ + *N*_2_). We seek to evaluate the entropy *S* = *k*ln*Ω* where *Ω* = *Ω*(*M,r*; *N*_1_,*N*_2_,*x*;*N*_0_) is the number of distinguishable configurations. Ideally we should require that this expression reduce to the exact limits wherever they are known (see the Introduction) and be independent of the manner of placing the squares and rods. We achieve these aims only partially.

### 2.1 Exact Results for Squares

First, we wish to establish the relation

$$\Omega (M,r;O,O,x;O)=2r^{\sqrt{M}}\;d=2.$$ (1)

From tiling theory [12] we know from Minkowski’s conjecture that there is at least one pair of hypercubes which share a complete (*d*−1)-dimensional face [13]. For squares this means that they share a complete edge. A little thought shows that the tiling must consist of infinitely long rows of squares. Each row can be translated one step *r* times to give *r* different configurations for the whole lattice. Since there are $\sqrt{M}$ rows each of length $\sqrt{M}$, there are $r^{\sqrt{M}}$ different configurations resulting from sliding the $\sqrt{M}$ rows relative to each other. The factor of 2 arises because the files can lie in either the *x* or *y* orientations. Edge effects are neglected.

In one dimension there are only *r* ways to cover the line with *r*-mers. There are *r* ways rather than one way because we can shift the whole close-packed row of rods one monomer at a time for a total of r times before we repeat a configuration. So, for *d* = 1 we obtain *Ω* (*M*,*r*;0,0,*x*;0) = *r*. The question naturally arises as to how many ways we can tile a three dimensional cubic lattice with cubes that are of size *r*^3^. The answer is essentially $3r^{M^{2/3}}$. To see this, first tile a two dimensional floor with cubes. There are $2r^{M^{1/3}}$ ways to do this. Next, imagine rows of cubes stacked perpendicularly to the floor. By moving these rows up and down r steps we obtain $r^{M^{2/3}}$ configurations. So in three dimensions

$$\Omega =3(2r^{M^{1/3}})\;(r^{M^{2/3}})\;d=3,$$ (2)

These formulas suggest the general expression

$$\Omega =K(d)r^{M^{(d-1)/d}}\;\text{for all}\;d,$$ (3)

where *K*(*d*) is of an order lower than *r^M^*^(d−1)/^*^d^*, as the formula for tiling hypercubic lattice with hypercubes. The entropy per hypercube is then

$$S/k=(\ln\Omega )/M=\ln K(d)/M+M^{-1/d}\ln r,$$ (4)

which vanishes as *M* → ∞. Thus *S* = 0 for the tiling of squares, or of cubes (the effective *Ω* is 1). This is an exact result.

### 2.2 Connection Between Flory-Huggins Lattice Statistics and the Poisson Distribution

#### 2.2.1 Exact Results for Rods Plus Solvent in *d* = 1

Consider first the number of ways, *Ω*, to place N_0_ monomers and *N_x_* rigid rods of length *X* (*x*-mers) on a one-dimensional lattice. *Ω* is obviously the same as the number of ways to permute *N*_x_ objects of one kind with N_0_ objects of another kind:

$$\Omega =(N_{0}+N_{x})!N_{0}!N_{x}!.$$ (5)

As we now show, this formula can also be obtained using the FH approach by calculating

$$\Omega =(\prod \nu_{j})/N_{x}!,$$ (6)

where *ν_j_*_+1_ is the number of ways to randomly place the (*j*+1)th rod (*x*-mer), given that the previous *j* molecules have been randomly placed. After the rods are all placed, the monomers can be placed in only one way. For *ν_j_*_+1_ we write

$$\nu_{j+1}=(N-xj){\left(S_{0}\right)}^{x-1},\;\;S_{0}=(N-xj)/(N-xj+j).$$ (7)

*N*−*xj* is the number of ways to place the first segment of the (*n* +1)th rod onto the lattice (it can go onto any of the *N*−*xj* empty sites) and *S*_0_*^x^*^−1^ is the probability that the *x*−1 consecutive neighboring sites are empty. *S*_0_ is the probability that a neighboring site is empty. To calculate *S*_0_, we write

$$S_{0}+S_{x}=1,\;S_{0}/S_{x}=(N-xj)/j,$$ (8)

where *S_x_* is the probability that the neighboring site is filled. Obviously *S*_0_ + *S*_x_ = 1. Since a step will be successful if one is a neighbor to a hole, and since a step will be unsuccessful if one is a neighbor to a rod, the ratio is equal to the ratio of the number of neighbors to holes (*N*−*xj*) to the number of neighbors to rods (*j*). Using Eq. (7) in Eq. (6) we obtain Eq. (5).

There are several useful observations at this juncture. First, for *d* = 1 the FH scheme gives the exact result. But one needs to use the surface fraction *S*_0_ rather than the more approximate volume fraction *V*_0_(*V*_0_ = (*N*−*xj*)/*N*). Second, since *S*_0_*^x^*^−1^ = exp(−λ(*x*−1)) with λ = −*lnS*_0_ we have a Poisson distribution [14] for the probability that a line segment of length *x*−1 is empty. As far as the statistics for placement of the (*ν* +1) th rod are concerned, the previous *n* rigid rods are all shrunk to points, so that we are dealing with Poisson statistics for points randomly sprinkled on a line. As we shall now see, these two features—the use of the surface fraction rather than the volume fraction, and the use of Poisson statistics—allow us to solve the two-dimensional problem.

#### 2.2.2 Extension of Poisson Statistics to Rods Plus Solvent in *d* = 2

Randomly place *N*_1_ horizontal thin lines of length *L*_1_ on a plane of area *A* ( $\sqrt{A}$ on a side). We now determine the probability that a line of length *x*, whose center of mass is located at random in the area and whose orientation is perpendicular to the previously placed lines, does not touch any of the lines. A perpendicular line of length $\sqrt{A}$ which spans the area will on average intersect $N_{1}L_{1}/\sqrt{A}$ horizontal lines so that the average separation between horizontal lines is $<x>\;=\sqrt{A}/(N_{1}L_{1}/\sqrt{A})=A/N_{1}L_{1}$. The Poisson distribution is obviously by exp (−*x*/< x >) = exp (−*xN*_1_*L*_1_/*A*). For the general case of a line of length *x* making an angle *θ* with the horizontal lines, we have exp (−*xN*_1_*L*_1_sin*θ*/*A*. Thus, the probability, *p*(*θ*), that the line can be placed without contacting any horizontal line is

$$p(\theta )=\exp(-\lambda x),\;\lambda =N_{1}L_{1}\sin\theta /A.$$ (9)

Notice that for *θ* = 0, *p*(*θ*) = 1 as expected, even for *x* → ∞ since the lines are infinitely thin.

Now consider *N*_1_ horizontal lines of length *L*_1_ and *N*_2_ vertical lines of length *L*_2_. The horizontal lines will cross a line of length $\sqrt{A}$, inclined at angle *θ*, $N_{1}L_{1}\sin\theta /\sqrt{A}$ times. This line also intersects the vertical lines $N_{2}L_{2}\cos\theta /\sqrt{A}$ times. Then, by calculating the average separation distance along the inclined line between the crossings, we obtain (< x > = 1/λ)

$$p(\theta )=\exp(-\lambda x),\;\lambda =N_{1}L_{1}\sin\theta /A+N_{2}L_{2}\cos\theta /A.$$ (10)

In the more general case where the number of lines *N*(*ϕ*) of length *L*(*ϕ*) are randomly placed with regard to their centers but inclined at an angle *ϕ*, we have

$$p(\theta )=\exp(-\lambda x),\;\lambda =\int A^{-1}N(\phi )L(\phi )\sin(\theta -\phi )d\phi .$$ (11)

Notice that *p*(*θ*) is a product of the *p*(*ϕ*)’s arising from each angle *ϕ*. Equation (9) is exact, and Eqs. (10) and (11) are exact if the lines are allowed to cross. If crossing is not allowed, then correlations are induced among the locations of the lines and the formulas are not exact.

#### 2.3.3 Results for Rectangles Plus Solvent in *d* = 2

Now instead of lines let us place *M* rectangles of dimensions *a* and *b* on the area, oriented so that *a* is the horizontal dimension. What is the interference encountered by a line of length *x* when it is placed in this field of rectangles? It is suggested that if we use an *A*′ given by *A*′ = *A*−*abM*, then all the above formulas hold. Just as in *d* = 1 where we shrunk the rigid rods to points, in *d* = 2 we shrink each rectangle to two lines—a horizontal line of dimension *a* and a vertical line of dimension *b*. In particular,

$$\begin{array}{c} p(h)=\exp(-xMb/A\prime ),\;p(\nu )=\exp(-xMa/A\prime ),\\ A\prime =A-abM, \end{array}$$ (12)

for lines placed horizontally or vertically. For a line placed at angle *θ*, we obtain Eq. (10), but with *A*′ replacing *A*.

Our final step is to calculate the probability for placing a rectangle in the unoccupied area *A*′. We use the hypothesis that this probability is equal to that for placing a line whose length and orientation is that of the diagonal to the rectangle. We have

$$\begin{array}{c} p=\exp(-(\sqrt{a^{2}+b^{2}})\;(\mathrm{Ma}\sin\theta +\mathrm{Mb}\cos\theta )/A\prime )\\ =\exp(-2abM/(A-abM)), \end{array}$$ (13)

where we have used $\sin\theta =b/(\sqrt{a^{2}+b^{2}})$ and *A*′ = *A*−*abM*. Let us compare this to the expression obtained from FH lattice statistics. After placing the corner site onto the lattice, we can place the column of vertical sites and the row of horizontal sites with a probability *p* (we use *A* = *N*)

$$\begin{array}{c} p=[(N-abM)/(N-abM+aM){]}^{b}\\ {}[(N-abM)/(N-abM+bM){]}^{a}. \end{array}$$ (14)

If we now write *p* as exp(ln*p*) and expand the logarithms we obtain Eq. (13) to first order in the argument of the exponential. *Thus we get concordance with Poisson statistics if, in the Flory-Huggins counting procedure when calculating the probability of placing down the (M+1)th rectangle, we ignore all sites other than the sites belonging to the two edges*! This is a surprising result which we now show leads to formulas for the entropy which reduce to the tiling results in the absence of solvent.

### 2.3 Entropy of a Mixture of Squares, Rods, and Solvent in *d* = 2

The implementation of the Flory-Huggins approach to the calculations of entropies for the general case is straightfoward but tedious. We will illustrate the essential features of the method by calculating separately in *d* = 2 the number of configurations for both a system of rigid rods plus solvent and for a system of squares plus solvent. The entropy for a two-dimensional system of rods plus squares plus solvent will be displayed and compared with the results of Boehm and Martire. Also, we propose a modification of the BM treatment which is less dependent on the order of placement.

#### 2.3.1 Rods Plus Solvent

To calculate the number of ways to place *N*_1_ rigid rods in the *x* orientation and *N*_2_ rigid rods in the *y* orientation on a square lattice of *N* = *x*(*N*_1_+*N*_2_)+*N*_0_ sites, we begin by placing the *N*_1_ molecules one at a time until they are all placed. This can be done in

$$\begin{array}{c} \Omega_{1}=(1/N_{1}!)\prod_{j=0}^{N_{1}-1}\left(\nu_{j}\right),\\ \nu_{j+1}=(N-xj)[(N-xj)/(N-xj+j){]}^{x-1} \end{array}$$ (15)

ways. Here *ν_j_*_+1_ is the number of ways to place the (*j*+1)th molecule in a field of j previously placed molecules. The first segment of this molecule, which we take to be the end segment, can be placed onto any of (*N*−*xj*) lattice sites. The probability of placing the second segment when the two segments lie in the *x* direction depends on the number of neighbors to holes in this direction compared to neighbors to rods in this direction. Using the ideas of Eq. (8), we calculate the probability that the second site is empty given that the first site is occupied to be (*N*−*xj*)/(*N*−*xj*+*j*). Proceeding in this way segment by segment, we obtain *Ω*_1_. We now calculate *Ω*_1,2_, the number of ways to place the *N*_2_ rods in the *y* direction given that the *N*_1_ molecules have been previously placed on the lattice:

$$\begin{array}{c} \Omega_{1,2}=(1/N_{2}!)\prod_{j=0}^{N_{2}-1}\left(\nu_{j}\right),\;\nu_{j+1}=(N-xN_{1}-xj)\\ {}[(N-xN_{1}-xj)/(N-xj+j){]}^{x-1}. \end{array}$$ (16)

The only new feature in this part of the calculation is that when determining the probability of successfully stepping in the *y* direction, each rod lying perpendicular to the direction of step contributes *x* sites, while each rod lying parallel contributes one site. *In the general case, a molecule contributes only those sites that are not shielded by other sites of the same molecule* (this statement is true for arbitrary dimension, arbitrary lattice type, arbitrary direction of stepping, and arbitrary stiffness). The total number of configurations is *Ω_r_* = *Ω*_1_*Ω*_1,2_. To evaluate *Ω_r_*, one can either rewrite the factors in terms of factorials and use Stirling’s formula, or convert the product to a sum by taking logarithms and use the formula for the integral of a logarithm [15]. Either way, we obtain

$$\begin{array}{l} \ln\Omega_{r}=-\sum_{k=1}^{2}[N_{k}\ln(N_{k}/N)+(N-(x-1)N_{k})\\ \;\ln(1-(x-1)N_{k}/N)]-N_{0}\ln(N_{0}/N). \end{array}$$ (17)

This result is *independent* of the order in which the rods are counted, as it should be if the FH procedure is self-consistent. Moreover, if we replace the upper limit on the summation by *d* and define *N_k_* to be the number of rods in orientation *k*, then Eq. (17) is valid for all dimensions [6].

#### 2.3.2 Squares Plus Solvent

For *M* squares, each of which cover *r*^2^ sites, we need to evaluate

$$\begin{array}{c} \Omega_{s}=(1/M!)\prod_{\nu_{j}},\\ \nu_{j+1}=(N-r^{2}j)\;[(N-r^{2}j)/(N-r^{2}j+rj){]}^{2(r-1)}. \end{array}$$ (18)

The factor on the left is simply the number of empty sites available to the first segment of the (*j*+1)th square. The term within the square brackets is the probability of successfully making a step along an edge of the square. This quantity, taken to the 2(*r*−1) power, is the probability that we can successfully complete two perpendicular edges. Equation (18) is the complete expression. The reason we do not add a factor for each of the remaining (*r*−1)^2^ monomers, as Boehm and Martire (following Herzfeld) do, is our hypothesis that the probability of placing a rectangle is equal to the probability of placing its diagonal line, which in turn is equivalent to placing two of its orthogonal edges. Equation (18) gives, after some algebra and integration,

$$\ln\Omega_{s}=-M\ln(M/N)-((2r-1)/r^{2})N_{0}\ln V_{0}+(2/r)\;(N_{0}+rM)\ln[(N_{0}+rM)/N].$$ (19)

We notice immediately that for no solvent the entropy is zero in the thermodynamic limit, which accords nicely with Eq. (4). Thus, our answer is exact in the *N*_0_→0 limit. Had we used a probability factor less than one for each of the (*r*−1)^2^ monomers in the interior of each square, the entropy for the case of *N*_0_ = 0 would have been negative. This supports our ansatz that in this problem a rectangle can indeed be represented by just two orthogonal edges. We will return to this point when we discuss the possibility of a close-packed amorphous glass of squares.

The only other exact result which is known for squares is the extremely dilute solution limit. In the absence of interaction energies, the mutual excluded volume between two particles is given by the second virial coefficient. To adapt this idea to our lattice model calculation, we must [in Eq. (18)] replace the *rj* term by *θrj*, and choose *θ* so that for *j* = 1 we obtain the correct number of ways to place the second molecule,

$$\nu_{1}=(N-r^{2})[N-r^{2})/(N-r^{2}+\theta r){]}^{2(r-1)}=N-(2r-1).$$ (20)

The RHS is obtained by subtracting from *N* the number of ways that the second molecule can overlap the first molecule. This equation yields *θ* = (3*r*−1)/2r, which varies from 1 to 1.5 as *r* varies from 1 to ∞. If we now use Eq. (18) with the *θrj* term replacing *rj*, we obtain

$$\begin{array}{l} \ln\Omega_{s}=-M\ln[M/N]-[(2r-1)/r^{2}]N_{0}\ln V_{0}+\\ {}[2(r-1)\;(N_{0}+\theta rM)/(r^{2}-\theta r)]\;\ln[V_{0}+\theta V_{r}/r], \end{array}$$ (21)

where

$$\theta =\theta_{0}-V_{r}(\theta_{0}-1)$$ (22)

and

$$\theta_{0}=(3r-1)/2r.$$ (23)

Equation (21) gives the known exact results for both small *V_r_* and for *V_r_* = 1. The linear interpolation of Eq. (22) is a reasonable assumption since *θ* varies between 1 and 1.5. One could easily improve upon Eq. (22) if the result for an intermediate *V_r_* were known through Monte Carlo simulation. In the equations that follow we will set *θ*_0_ = 1, but we note here that improvement of the lattice results through the virial approach is always possible.

#### 2.3.3 Rods, Squares, and Solvent

We now can solve the problem of mixtures of rods plus squares plus solvent (or holes). The algebra is considerable: we quote the results. If we place the rods first and then the squares, we obtain

$$\begin{array}{l} \ln\Omega_{r,s}(M,r;N_{1},\;N_{2};\;N_{0})=\\ +\sum [(r-1)/r]\;[N-(x-1)N_{i}]\;\ln[1-(x-1)N_{i}]\\ +(1/r)\sum [N-(x-1)N_{i}-(r^{2}-r)M]\\ \ln[1-((x-1)N_{i}+(r^{2}-r)M/N]\\ -[(r-1)/r{]}^{2}\;[N-xN_{x}]\;\ln[1-xN_{x}/N]\\ -[(2r-1)/r^{2}]\;[N-xN_{x}-r^{2}M]\;\ln[1-(xN_{x}+r^{2}M)/N]\\ -\sum N_{i}\ln[N_{i}/N]-M\ln[M/N], \end{array}$$ (24)

where the summation is over both orientations and *N_x_* = Σ*N_i_*. If we place the squares first and then the rods, we obtain

$$\begin{array}{l} \ln\Omega_{s,r}\;(M,\;r;\;N_{1},\;N_{2};\;N_{0})=\\ +\sum [N-(x-1)N_{i}-(r^{2}-r)M]\\ \ln[1-((x-1)N_{i}-(r^{2}-r)M/N]\\ -[2(r-1)/r]\;[N-(r^{2}-r)M]\;\ln\;[1-(r^{2}-r)M/N]\\ -[(r-1)/r{]}^{2}\;[N-r^{2}M]\;\ln[1-r^{2}M/N]-N_{0}\;\ln[N_{0}/N]\\ -\sum N_{i}\;\ln\;[N_{i}/N]-M\ln[M/N]. \end{array}$$ (25)

Each of these equations reduces to the rigid rod case, Eq. (17), when there are no squares, and reduces to the square case, Eq. (19), when there are no rods. Additionally, each reduces to the exact results obtained from tiling theory, ln*Ω* = 0, when there are no rods or solvent. Also, if we wished, we could introduce *θ*’s as above which would give us the exact results for dilute solution. However, the results do depend on the order of placement, and this is a defect in the FH procedure. The crucial question is, how different?

We attempt to resolve this discrepancy by placing one square in a field of *N*_1_ rods parallel to the *x* direction, and *N*_2_ rods parallel to the *y* direction, and *N*_0_ solvent molecules. This should give the same statistics as placing the square first and then adding the rods. Since we are confident that we know the statistics for adding the rods into a field containing one square, we can use this result for the statistics of placing one square after all the rods are placed. We obtain

$$\begin{array}{c} \nu_{s,\;N_{1},\;N_{2}}=\nu_{N_{1},\;N_{2},s}=\\ (N-xN_{1}-xN_{2})\;[(N-xN_{1}-xN_{2})/(N-(x-1)N_{1}){]}^{\left(r-1\right)}\\ {}[(N-xN_{1}-xN_{2})/(N-(x-1)N_{2}){]}^{\left(r-1\right)}\\ {}[(N-xN_{1}-xN_{2})N/(N-(x-1)N_{1})(N-(x-1)N_{2}){]}^{\left(r-2\right)}. \end{array}$$ (26)

Boehm and Martire [3] would use the same first three terms, but instead of our last term they would have

$$[(N-xN_{1}-xN_{2})/{\left(N-(x-1)(N_{1}+N_{2})\right]}^{{\left(r-2\right)}^{2}}.$$ (27)

The two expressions account for the probability of filling in the (*r*−1) segments after the corner segment and the two edges are first placed. They are the same only when the rods are all aligned in one direction. Still, in both cases the value of the last term is much less than the value 1 which we used above in deriving Eqs. (24) and (25). The general expression for filling in the (*r*−1) segments of the (*j*+1)th square in a field consisting of *N*_1_, *N*_2_ rods and *j* squares on a lattice of *N* sites, is given by BM as

$$[(N-x(N_{1}+N_{2})-jr^{2})/{\left(N-x(N_{1}+N_{2})-j(r^{2}-1))\right]}^{{\left(r-1\right)}^{2}}.$$ (28)

A natural generalization of our fill-in term is

$$\begin{array}{c} \left[(N-x(N_{1}+N_{2})-jr^{2})/(N-x(N_{1}+N_{2})-j(r^{2}-1)\right)\\ +\;(x-1{)}^{2}N_{1}\;N_{2}/N{]}^{{\left(r-1\right)}^{2}}. \end{array}$$ (29)

which differs from the BM term only by the (*x*−1)^2^ *N*_1_*N*_2_/*N* term in the denominator.

Using these expressions, we can derive alternative formulas for the entropy of mixtures of squares, rods, and solvent. For the Boehm-Martire version we simply add the factors obtained from Eq. (28) for the fill-in process, and append the result to Eq. (24) to obtain

$$\begin{array}{l} \ln\Omega_{r,s}\;(\text{BM})=\ln\Omega_{r,s}+[(r-1)/r{]}^{2}\;(N-xN_{x})\\ \ln\;(1-xN_{x}/N)-[(r-1)/(r+1)]\;(N-(x-1)N_{x})\\ \ln\;(1-(x-1)N_{x}/N)\\ -[(r-1)/r{]}^{2}\;(N-xN_{x}-r^{2}M)\;\ln\;(1-(xN_{x}+r^{2}M)/N)\\ +[(r-1)/(r+1)]\;(N-(x-1)N_{x}-(r^{2}-1)M)\\ \ln\;(1-((x-1)N_{x}/N-(r^{2}-1)M)/N), \end{array}$$ (30)

and,

$$\begin{array}{l} \ln\Omega_{s,r}\;(\text{BM})=\ln\Omega_{s,r}-[(r-1)/r{]}^{2}(N-r^{2}M)\\ \ln(1-r^{2}M/N)+[(r-1)/(r+1)](N-(r^{2}-1)M)\\ \ln(1-(r^{2}-1)/M/N). \end{array}$$ (31)

For the modified Boehm-Martire formulas (MBM) which use Eq. (29) we obtain

$$\begin{array}{l} \ln\Omega_{r,s}\;(\mathrm{MBM})=\\ \ln\Omega_{r,s}+[(r-1)/r{]}^{2}(N-xN_{x})\ln(1-xN_{x}/N)\\ -[(r-1)/(r+1)](N-(x-1)N_{x}+(x-1{)}^{2}N_{1}N_{2}/N)\\ \ln(1-((x-1)N_{x}-(x-1{)}^{2}N_{1}N_{2}/N)/N)\\ -[(r-1)/r{]}^{2}(N-xN_{x}-r^{2}M)\ln(1-(xN_{x}+r^{2}M)/N)\\ +[(r-1)/(r+1)](N-(x-1)N_{x}+(x-1{)}^{2}\\ N_{1}N_{2}/N-(r^{2}-1)M)\ln(1-((x-1)N_{x}-(x-1{)}^{2}\\ N_{1}N_{2}/N+(r^{2}-1)M/N), \end{array}$$ (32)

and

$$\begin{array}{l} \ln\Omega_{s,r}\;(\mathrm{MBM})=\\ \ln\Omega_{s,r}-[(r-1)/(r+1)](N+(x-1{)}^{2}N_{1}N_{2}/N)\\ \ln(1+(x-1{)}^{2}N_{1}N_{2}/N^{2})-[(r-1{)}^{2}/r{]}^{2}(N-r^{2}M)\\ \ln(1-r^{2}M/N)+[(r-1)/(r+1)](N+(x-1{)}^{2}\\ N_{1}N_{2}/N-(r^{2}-1)M)\;\ln\;(1+((x-1{)}^{2}\\ N_{1}N_{2}/N-(r^{2}-1)M)/N). \end{array}$$ (33)

Let us now summarize our results. We have presented three pairs of formulas for the entropy of a mixture of rods, squares, and solvent. For each pair, the entropy depends on the order of placement of the rods and squares. Consequently, for the special cases of rods plus solvent only, or squares plus solvent only, the entropies are equal within each pair. Also, for rods plus solvent only, all six equations are identical and give the usual result. However for squares plus solvent only, the Boehm and Martire (BM) and Modified BM (MBM) are identical while the DYG approximation gives a different result. The first pair, Eqs. (24) and (25) has the virtue of reducing to the exact tiling result when there are only squares. The second pair, Eqs. (30) and (31) is the Boehm-Martire result. The third pair (MBM), Eqs. (32) and (33) is similar to the BM result but uses Eq. (29) for the probability of placing the (*r*−1)^2^ fill-in segments onto the lattice after one corner segment and the two connecting edges have been placed. The BM calculation uses the same probability as MBM, but with the last term of the denominator missing. It is obvious that our two estimates of the entropy, DYG and MBM, always bracket the Boehm-Martire entropy.

**Fig. 1:**
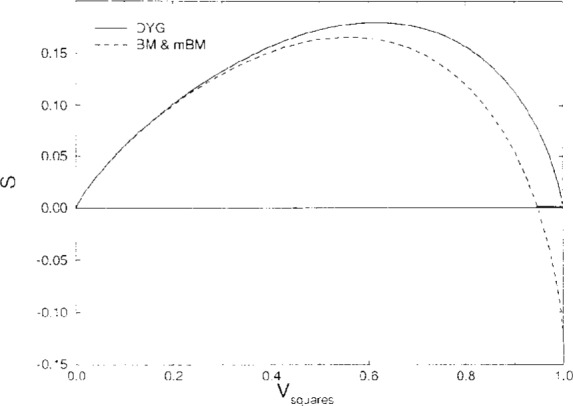
Comparison of the entropy of square-solvent mixtures in two dimensions for squares of aspect ratio (edge length to thickness) 3. The upper, DYG, curve reduces to the tiling result for no solvent. The lower curve gives the BM and MBM results which superpose when there are no rods. See text.

Figure 1 shows the three entropy predictions for squares plus solvent in *d* = 2. The only qualitative difference between the curves is in the high *V_r_* region where our DYG expression gives positive entropy over the whole range of concentration, while the BM and MBM expressions become negative. At first sight it could be argued that the DYG expression is the more accurate one at high density, since for a density of 1 it gives the correct tiling result, while the BM and MBM predictions of negative entropy are clearly wrong. However, in the next paragraph we argue that the BM and MBM entropies, suitably modified by replacing the *S* < 0 part the curves with the line *S* = 0 may give better estimates of the entropy for amorphous systems. Moreover, the crossing points give estimates of the close packed amorphous density.

Consider a system of rigid rods plus solvent. Our rigid-rod statistics correctly predict a close-packed amorphous state of less than unit density for *d* ≥ 2. One simply cannot pack rods in random orientation when the rod density is high, and our rigid rod statistics predict this by giving zero entropy at a density less than one. The reader can convince himself of this by trying to pack pencils randomly at high density; it can’t be done. This simple physics was recognized by Onsager as the basis for the isotropic to nematic phase transition in liquid crystals. This so called “entropy catastrophe” carries over to semiflexible polymer molecules and is the basis for the entropy theory of glasses [16], [17]. The Kauzmann paradox was resolved along these lines [16]. The fact that our rigid rod statistics, and their modification for semiflexible polymers predict (semiquantita-tively) the experimental results for both liquid crystals and polymer glasses, means that they are reasonably accurate [17], and the fact that the entropy catastrophe is real means that our formula for the entropy should approach zero at a finite density [18], as our formulas indeed do. Perhaps, then, the entropy for squares becoming zero, as in Fig. 1, for a density less than one is not merely an artifact of the calculation but is an indication of a close-packed amorphous packing of squares. So, it is possible that there is an entropy catastrophe for squares as well as for rigid rods in *d* = 2 and this is presaged by the BM and MBM estimates. One thing we can be sure of is that in *d* = 3 the problem is much more acute. A close packed amorphous phase of squares in *d* = 3 resembles a house of cards, and for this case as well as the case of rods in *d* = 3 the only way we can achieve high density is to align the squares parallel to one another just as one needs to align the rods parallel to one another to achieve higher density in that case. We turn our attention to the three-dimensional problem in the next section.

**Fig. 2:**
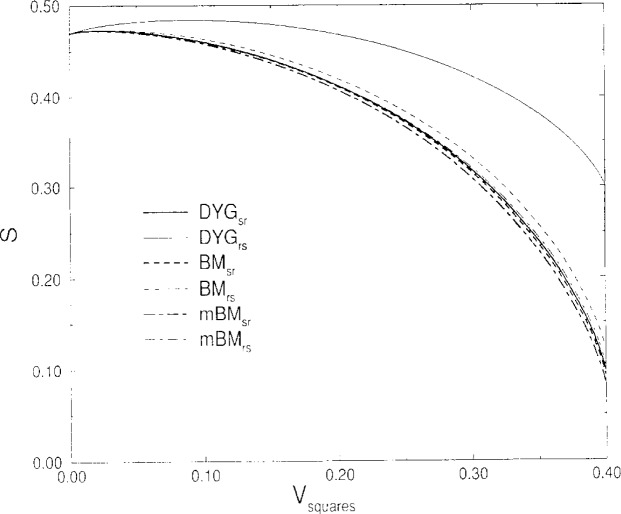
These figures show the extent for which the three methods of counting give different results depending on the order of placement of rods and suares. The plot is of entropy vs volume fraction of squares *x* = *r* = 3. The volume fraction of rods is fixed at 0.6. The Boehm-Martire curves are less different than those which incorporate the tiling results of this paper, but more different than the modified version (MBM). The difference between the MBM curves is about 2/3 of that between the BM curves.

Figure 2 compares the three pairs of curves for the entropy of a mixture of rods, squares, and solvent where the rod length *x* equals the edge length *r* of the square, and (excluding solvent) 30 % by volume of the material is rods and 70 % is squares. A surprising and discouraging result is that the DYG calculation depends more on the order of placement than does the Boehm-Martire calculation. The MBM calculation, however, does better. Obviously, we have not yet solved the “order of placement problem.”

### 2.4 Entropy of a Mixture of Rods, Squares and Solvent in 3-Dimension

In this section we generalize the results of the previous section to calculate the entropy of a mixture of rods, squares and solvent in *d* = 3. In Sec. 2.3.1 we already calculated the entropy of rods plus solvent for arbitrary dimension *d*. Thus, we begin with squares plus solvent in *d* = 3, and then combine the two results as in the previous section.

#### 2.4.1 Squares Plus Solvent in *d* = 3

Instead of giving the derivation in detail, we describe only those parts of the derivation which are unfamiliar to one who has used only the Flory version of the FH lattice model, or who has not used orientation dependent statistics.

As in Eqs. (10) and (11), we need to consider surface fractions rather than volume fractions. The probability of placing a specified object into the lattice depends upon the orientation and shape of the object relative to the orientations and shapes of the previously placed objects. For squares in *d* = 3 there are three orientations in which to place them (six orientations for rectangles). The number of ways to place a square is equal to the number of ways to place one segment of the square times the probability that the sites required to accommodate the remaining (*r*^2^−1) segments of the square are empty. To estimate this probability, we place these segments one at a time and assume that the probability is a product of the individual probabilities of placing the individual segments. These individual probabilities are conditional probabilities. We must now consider the probability of placing the kth segment, given that the previous (*k*−1) segments have been placed. In building up a square we first place down a corner in as many ways as there are empty lattice sites, and then we build up the two connecting edges. If the number of empty lattice sites is *N*_0_(*j*), then the probability of placing a contiguous segment in the *x* direction is *N*_0_(*j*)/(*N*_0_ + number of neighbors to polymer). The hypothesis here is, as before, that the ratio of the probability of a successful step to the probability of an unsuccessful step equals the number of neighbors to holes divided by the number of neighbors to polymer [(see Eqs. (10), (11)]. Importantly, this ratio is dependent on the orientation of the step. If we are stepping against a square which lies perpendicular to our step direction, then there are *r*^2^ interference sites due to that square, and if we are stepping against a square which lies parallel to our step direction, then there are r interference sites. The number of neighbors to polymer in the above expression for the number of ways to place a segment, is the sum of all the interferences arising from the previously placed (*j*−1) molecules. In the general case of a solid object of arbitrary shape, the interference is given by the projected area in the direction of the step.

We now build up each edge one segment at a time until both edges are completed. In our derivations we stop here [(see Eqs. (28), (29)] and our accompanying discussion. However, one could argue that we should continue and include in our product of probabilities the conditional probability that the corner segment diagonally opposite to the first placed corner segment is empty. After this is estimated the remaining (*r*−1)^2^−1 segments can be filled with probability 1. Obviously there is room for improving our estimate of the probability that a particular contiguous set of lattice sites are all simultaneously empty (see Sec. 3.).

The formula for squares plus solvent in *d* = 3 is straightforward, but tediously derived. The result is

$$\begin{array}{c} \ln\Omega_{s}=-((2r-1)/r^{2}))\;N_{0}\ln(N_{0}/N)-\sum M_{i}\\ \ln(M_{i}/N)+\sum [N/r-(r-1)(M-M_{i})]\\ \ln[1-(r^{2}-r)(M-M_{i})/N]. \end{array}$$ (34)

A treatment parallel to our treatment of the *d* = 2 problem reqires us to do the analogue of the BM and MBM treatments as well; this will be deferred to another publication.

For rigid rods, the orientation dependent lattice statistics predict that an isotropic phase can exist in equilibrium with an ordered phase for *d*≥ 2 in the absence of energetics. Similarly, for squares in *d* ≥ 3 an isotropic phase can coexist in equilibrium with an ordered phase in the absence of energetics. The straightforward way to determine the phase diagram is to equate chemical potentials, with each orientation denoting a separate species, but when there are equal numbers of squares in the three orientations the calculation is simplified. For the isotropic phase, *M*_1_ = *M*_2_ = *M*_3_ = *M*/3, and for the ordered (oriented) phase *M*_2_ = *M*_3_ = 0, *M*_1_ = *M*. By equating the chemical potentials of the two phases obtained from Eq. (34) evaluated with these substitutions, we obtain the equilibrium volume fractions of the two phases. Note that in the isotropic phase the three species [19] of squares occur in equal numbers, so that only one chemical potential is needed, and in the ordered phase there is only one species present, so again only one chemical potential is needed. The conditions for equilibrium are then

$${\mu_{0}}^{\text{isotropic}}={\mu_{0}}^{\text{ordered}},$$ (35)

and

$${\mu_{M}}^{\text{isotropic}}={\mu_{M}}^{\text{ordered}}.$$ (36)

An alternative and more accurate procedure, which is left to future work, is to define an order parameter *M*_1_/*M*, with *M*_2_ = *M*_3_, and then look for minima in the free energy vs order parameter curves.

**Fig. 3:**
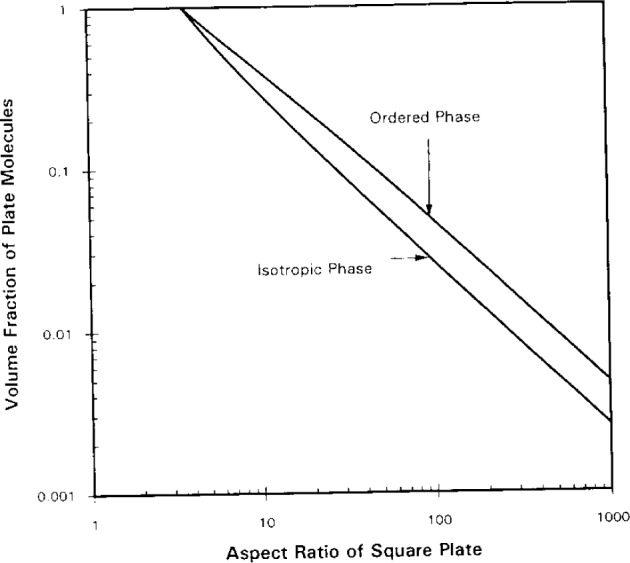
Entropy alone is sufficient to cause a phase transition for a plate plus solvent system in three dimensions. The region below the lower curve of a pair indicates a pure isotropic phase, while the region above the upper curve indicates a pure layered phase. Between the two curves lies the two-phase region. The amount of each phase is determined by the lever rule. The inability to pack at random at high density forces the plates to become parallel as we increase the density past the close-packed amorphous density. An equation which bisects the two-phase region is *rV_r_* = 3.55. For comparison, the equation for rigid rods is approximated by *x*V*_x_* = 3.8. See Fig. 4.

Figure 3 shows the results on a double-logarithmic plot for squares in *d* = 3. The region beneath the curves denotes the isotropic phase, while the region above the curves denotes the ordered phase. The two phase region exists between the two curves, with the amount of each phase determined by the lever rule applied to a vertical line connecting the two curves. The lower line gives the concentration of the isotropic phase and the upper line that of the ordered phase. The curves come together at an edge length of 3.55. Below this value the phase is always isotropic. The curve given by the formula

$$rV_{r}=3.55$$ (37)

where *V_r_* is the volume fraction of squares, comes close to bisecting the two phase region, and gives a good estimate of the concentration at which half the material is in each phase. One way of estimating the *V_r_* for which interferences among randomly-oriented squares first becomes a problem as the concentration is increased, is to build a house of cards in which each cube of volume *r*^3^ is made by lining the six sides with squares. This gives the formula *rV_r_* = 3, which reproduces the bottom curve of Fig. 3 to within 12 % (this statement is valid for *r* ≥ 5, and the equation is exact for *r* = 8).

**Fig. 4:**
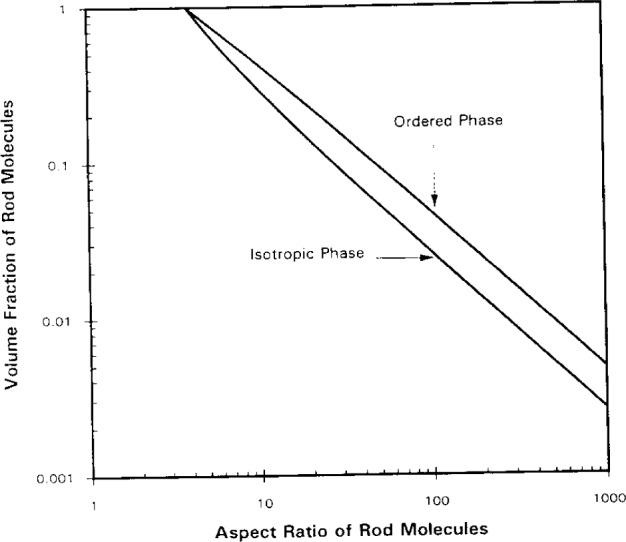
Entropy alone is sufficient to cause a phase transition for rigid-rod plus solvent systems in *d* ≥ 2 dimensions. The curves for *d* = 3 are shown. The curves for *d* ≥ 3 (not shown are displaced to the right. The location of the critical molecular weight, *x*_c_ below which there is no transition is given by *x*_c_ = 2.9*d*^1/4^. The bisector of each pair of curves is given by *xV_x_* = *x*_c_.

For comparison purposes, in Fig. 4 we have plotted the analogous curves for rigid rods (of length *x*) for three dimensions. Remarkably, the pair of curves for *d* = 3 almost superpose those for squares. Except for low *x* and *r* the lower curve is coincident to within 1/4 %, while the upper curve is 1 % higher than the corresponding curve for squares. The curves meet at a critical concentration of unity and a critical rod length, *x*_c_, below which on the isotropic phase exists. We have also calculated the phase diagrams for other dimensions. For *x*_c_ values given by *x* = 3.4, 3.8, 4.1, 4.35, 4.55, 4.85, 5.1, 5.3 corresponding to d values given by *d* = 2, 3, 4, 5, 6, 8, 10, 12. The value of *x*_c_ below which only the isotropic phase exists is well represented by the following equation

$$x_{c}=2.9d^{1/4}.$$ (38)

**Fig. 5:**
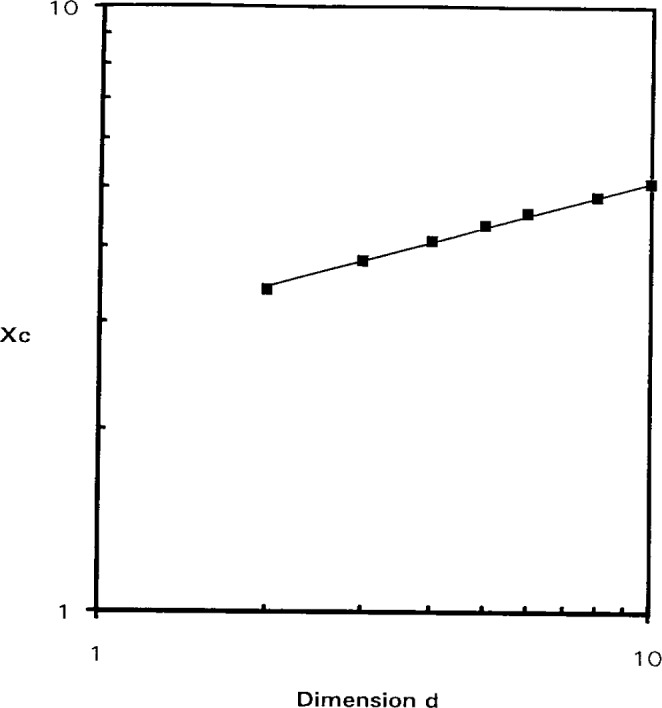
A comparison of the relation *x*_c_ = 2.9*d*^1/4^, which is the displayed curve, with the actual calculated points.

Figure 5 displays this equation along with the data points. Nemirovski, Huston, Graham, and Freed [20], using more accurate statistics, give an *x*_c_ value of 4.036 for *d* = 3. This suggests that perhaps the coefficient in Eq. (38) is 3 rather than 2.9 since this would result in an *x*_c_ value of 3.95.

#### 2.4.2 Squares Plus Rods Plus Solvent in 3-d

For squares placed first, then rods in *d* = 3, we obtain

$$\begin{array}{c} \ln\Omega_{s,r}=[(r-1)/r{]}^{2}(N-r^{2}M)\;\ln(1-r^{2}M/N)\\ -N_{0}\;\ln(N_{0}/N)-\sum M_{i}\ln(M_{i}/N)-\sum N_{i}\;\ln(N_{i}/N)\\ +\sum (N-(r^{2}-r)(M-M_{i})-(x-1)N_{i})\;\ln(1-((r^{2}-r)(M-M_{i})\\ +(x-1)N_{i})/N)-\sum [N(r-1)/r-(r-1{)}^{2}(M-M_{i}]\\ \ln(1-(r^{2}-r)(M-M_{i})/N). \end{array}$$ (39)

For rods placed first, then squares, we obtain

$$\begin{array}{c} \ln\Omega_{r,s}=-[(r-1)/r{]}^{2}(N-xN_{x})\;\ln(1-xN_{x}/N)\\ -\sum M_{i}\ln(M_{i}/N)-\sum N_{i}\ln(N_{i}/N)-[(2r-1)/r^{2}]\\ N_{0}\;\ln(N_{0}/N)+[(r-1)/r]\sum (N-(x-1)N_{i})\\ \ln(1-(x-1)N_{i}/N)+r^{-1}\sum [N-(r^{2}-r)(M-M_{i})\\ -(x-1)N_{i}]\;\ln(1-((r^{2}-r)(M-M_{i})+(x-1)N_{i}/N). \end{array}$$ (40)

These formulas reduce to the correct limits for *M* = 0 or for *N*_1_ = *N*_2_ = 0. They have been written in a form that suggests that for higher dimension we merely replace the upper limit on the summation by *d*; however, we have not proved this.

### 2.5 Cubes Plus Solvent in *d* = 3

For completeness, we display the result for cubes in *d* = 3:

$$\begin{array}{c} \ln\Omega_{c,3}/N=-([(r-1{)}^{3}+1]/r^{3})V_{0}\;\ln\;V_{0}\\ -[V_{r}/r^{3}]\ln[V_{r}/r^{3}]+[(r-1)/r{]}^{2}[1-(r-1)V_{r}/r]\\ \ln[1-(r-1)V_{r}/r]. \end{array}$$ (41)

Thus we now have formulas for rods in one, two, and three dimensions, squares in two and three dimensions, and cubes in three dimensions, as well as rods plus squares in two and three dimensions.

## 3. Discussion of Results

We have tried to develop a logical procedure for the generalization of the ideas of Flory-Huggins to treat molecules other than flexible polymers. Some time ago, Flory adapted the FH methods to rigid rods in order to treat liquid crystals [4, 21]. His treatment was a mix of lattice and continuum methods. Di Marzio, using the more accurate surface of Huggins fraction rather than the Flory volume fraction, obtained a quantitative improvement of the statistics [6]. Shih and Alben [9] (SA), Herzfeld [10], and Boehm and Martire [3] generalized the treatment to include plate like molecules, rectangular parallelepipeds and mixtures of plate-like molecules and rods.

The treatment of mixtures of rods and squares by Boehm and Martire shows that the FH procedure itself is breaking down since different sequences of placing the molecules give different results. This paper is an attempt to put the FH procedure on a firmer foundation while maintaining the simplicity of the method. It is useful to have a method of such wide applicability as is displayed in this work, and especially in the work of Herzfeld [10], if in fact we can believe the results. More accurate counting schemes are available [8] but they are not simple and involve considerable mathematical sophistication.

Since the FH scheme is approximate, it is appropriate to try to adjust the scheme so that it gives the correct results for those few cases for which we know the results exactly. Tiling theory [12] gives exact results for squares (no solvent or rods) and cubes. Also, we know the exact results for the (extremely) dilute solution range. Some of our final results incorporate these exact limits. Eq. (21) provides an example for the case of squares plus solvent.

It was also appropriate to examine the approximations of the FH model to see if they could be improved. The lattice theory gives exact results for the case of rods oriented in one direction, so we examined that case. There seem to be two critical assumptions. The first assumption is that in estimating the probability that a length of *x* contiguous segments be simultaneously empty we use the Poisson distribution. In one dimension it is not really an assumption since points randomly sprinkled on a line obey Poisson statistics, and consequently the probability of finding a free run of length *x* is given by the exponential distribution. This distribution is automatically obtained if we assume that the probability for placing *x* contiguous sites is given by the product of the probabilities for placing one site, as is assumed in the lattice model. The second assumption is that we use the Huggins surface fraction rather than the Flory volume fraction. This assumption is justified in the text.

It was then shown how, for rigid rods in higher dimensions, the use of a Poisson distribution is quite natural. The probability of placing a line of length *x* at random in a field of oriented rods is itself orientation dependent [see Eqs. (9)–(14)]. As shown previously, the probability *p* of succesfully taking one step is given by an orientation dependent surface fraction. *p* is called a surface fraction because only the sites on the surfaces of previously placed molecules interfere with the placement of segments (all but the first segment) of the *n* th molecule (see text). An understanding of why the FH statistics and their orientation-dependent generalizations are so accurate for linear polymers is thereby obtained.

In an important paper [10], Herzfeld applied orientation dependent lattice statistics to treat parallelepipeds which include plates as well as fat rods. Boehm and Martire applied the method to a mixture of square plates and rods to solve a two dimensional adsorption problem [3]. They discovered that the result depended on the order of placement of the rods and squares. This is disconcerting because then one does not know which result to believe. So we have examined the problem anew for the case of a mixture of non-energetic plates, rods and solvent, which is perhaps the simplest case in which the problem of order-dependence presents itself.

**Fig. 6:**
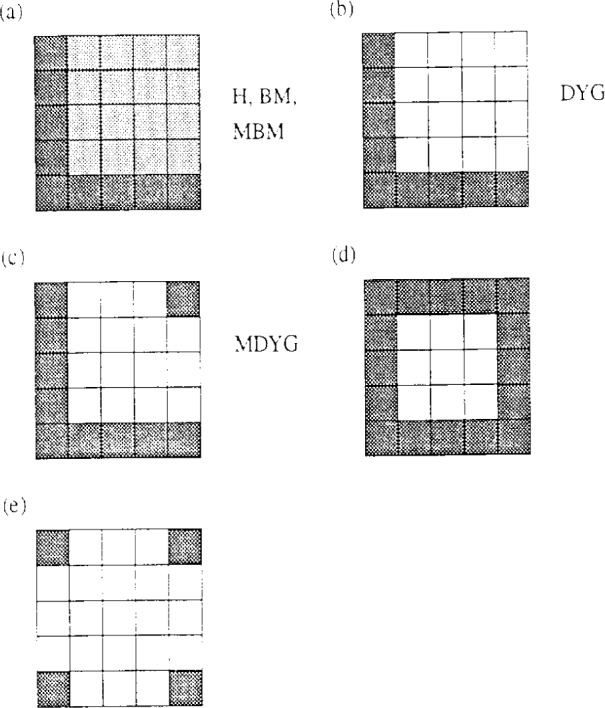
Five different ways of calculating the probability of placing a square of 5^2^ segments into a field of like squares. One easily verifies that in any of the figures the probability of simultaneously placing the darkened monomers is equal the probability of placing the square. See text.

Generalization to the placement of plates rather than lines (into a field of like objects) was accomplished by arguing that placing a plate can be done with the same probability as placing selected sets of lines contained in the plate. It was argued that placing a plate against other like plates is equivalent to placing two of its perpendicular edges, or equivalently, its diagonal (see text). The probability of placing a line is orientation dependent. In general, the central problem, which has not been solved, is given that the probability of placing a straight line of length *x* in orientation *Ω* is exp (−*x*/< *x*(*Ω*)), where < *x* (*Ω*) is known, what is the probability of placing a given shaped area (volume) at a given orientation? In Fig. 6, we show 5 figures that describe various approaches to the problem. Figure 6a describes an adaptation of the SA statistics to squares by Boehm and Martire. One first lays down the corner site, then each of the two edges, then the remaining (*r*−1)^2^ sites. Figure 6b describes our first approach where we lay down the corner site and the two edges with identical probabilities just as SA and BM do, but lay down the remaining (*r*−1)^2^ sites with a probability equal to 1. Our justification for this was first that it gives the correct result for the case of complete tiling, and second our argument that the probability for laying down a square is the same as that for laying down a diagonal to the square (see text). However, in the Theory Section, we argue that the SA, BM procedure is treating the close packed *amorphous* region more correctly, while our treatment may be treating the high density crystalline region more correctly.

Figure 6c is an attempt to solve the problem by arguing that the probability of placing the square is equal to the probability of simultaneously placing the two edges and the one site on the opposite diagonal. This is certainly true since placing these sites in a field of squares implies that one can place the square, but this is not the same as saying that the probability is equal to the probability of placing the two edges *times* the probability of placing the diagonally opposite corner site. Similarly, the probabilities of simultaneously placing the perimeter sites, Fig 6e, or the four corner sites, Fig. 6d, is equal to the probability of placing the whole square, but this does not mean that we get the correct answer by multiplying *independent* probabilities. So the fundamental idea that the probability of simutaneously placing a selected set of sites is the product of the probabilities for placing the individual sites fails for objects other than linear chains. This being the case, it is probably best to choose the factor for the (*r*−1)^2^ fill-in sites to fit a Monte Carlo calculation which is to be taken as the exact answer.

Another approach taken [see Eqs. (28), (29)] was to use the fact that there should be no differece in results (if we place squares first, then rods or conversely) as a way of deciding the expression for the probability of filling-in the last (*r*−1)^2^ sites. The results, Eqs. (32), and (33) are always smaller than the corresponding BM expression. Thus, the BM entropy lies between our two estimates.

We began our investigation with the confidence that knowing the exact answer for the tiling of squares would allow us to select which of the three possibilities was the correct one. However, we no longer believe this. An isotropic distribution of plates in *d* = 3 shows an entropy catastrophe just like that observed for rigid rods in *d* = 3. In our view, this is real and is a basis for predicting an entropy-driven phase transition from disordered to ordered plates just as the entropy catastrophe for rods (originally pointed out by Onsager) is the basis for an entropy-driven isotropic to nematic phase transition in rigid rod systems. Further, this entropy catastrophe is the basis for predicting glass formation in isotropic plate systems, in isotropic rigid rod systems, and finally in semiflexible polymer systems [17]. Our lattice calculation is obviously picking up the amorphous close packed state, and we cannot expect that the tiling results would apply in the close packed amorphous region.

In discussing the differences between the different calculations we have perhaps deemphasized the similarities, which we now enumerate. First, relatively simple calculations can be used to calculate the entropy of collections of molecules with complex shapes. Simple generalizations of Refs. 6, and 10, and this paper can be used for this purpose. Thus, the thermodynamic properties of these complicated systems can be easily obtained. Second, for squares, the methods all use the same statistics for placing the initial corner segment and the segments of the two edges connecting this corner. The methods differ only in the assignment of probabilities for adding the (*r*−1)^2^ segments to complete the square. The three methods used each have a particular advantage. The probability used by SA, Herzfeld, and BM is the most intuitive. The MBM method of Eqs. (28) and (29) combines this intuition with the requirement that placing one square after *N*_1_ rods in the *x* orientation and *N*_2_ rods in the *y* direction have been placed gives the same result as placing the square first, then adding the rods. This is the best we could do in our attempt to solve the order of placement problem. Finally, assigning a probability 1 for placing each of the (*r*−1)^2^ fill-in segments gives the correct tiling result for squares. The three-dimensional results of this paper use this assignment. What is needed is a Monte Carlo calculation of the number of configurations for a density in the region of close packed amorphous density. This would allow us to decide among the three possible choices.

Generalization of lattice results to a continuum of angles is of course possible along the lines of such generalizations made previously for the rigid rod problem [6]. In the rigid rods on a lattice problem, a coordination number existed which gave the same results as a continuum treatment [6]. We suspect the same to be true for plates. In view of this fact, and the fact that the lattice model is exact in both *d* = 1 and *d* = ∞, we believe the continuum treatment gains little.

Recently, Li, Freed, and Nemirovski have developed an expansion technique for for arbitrary shaped molecules on a lattice, and have applied it to squares [22].

In this paper we have avoided discussing energetics in order to focus on the entropic part of the problem. The introduction of energetics results in many additional phases and allows us to attempt a classification of the possible phases of discotic type molecules along the lines of that for liquid crystals and soaps. If we associate the long axis of the rigid rod with the perpendicular to the plane of the plate-like molecule, then it is obvious that every phase for liquid crystals has its analogue for plate-like systems. Proceeding in the other direction, we see that the columnar phase of discotics must have its analogue for liquid crystals. Thus, the subject of phase transitions in plate-like systems is potentially as rich a subject area as phase transitions in liquid crystals and soaps. An obvious difficulty in introducing interaction energies is the non-applicability of van der Waals forces for large molecules; the dispersion forces are generally of longer range than one obtains by using simple additivity of the pair potentials [23]. This can be a large effect. Finally, Brownian motion, which allows the molecules to sample space, is greatly reduced for large molecules. Plates, which can consist of thousands of atoms held rigidly count as very large molecules. Thus, the rate at which phase space is being sampled by molecular bombardment of the plates by solvent molecules is much slower than for liquid crystals or polymer systems; consequently, we should expect that the attainment of true equilibrium in these systems may be severely compromised.

## References

[1] Viai RA, Ishii H, Giannelis EP (1993). Chem Mat.

[2] Chandrasekhar S (1992). Liquid Crystals.

[3] Boehm RE, Martire DE (1992). Liquid Crystals.

[4] Flory PJ (1956). Proc Roy Soc.

[5] Onsager L (1949). Ann NY Acad Sci.

[6] Di Marzio EA (1961). J Chem Phys.

[7] McCrackin FL (1978). J Chem Phys.

[8] Freed KF, Bawendi MG (1989). Phys J Chem.

[9] Shih CS, Alben R (1972). J Chem Phys.

[10] Herzfeld J (1982). J Chem Phys.

[11] Kasteleyn PW (1961). Physica.

[12] Stein SK (1974). Amer Math Monthly.

[13] Minkowski H (1907). Diophantische Approximationen.

[14] Feller W (1957). An Introduction to Probability Theory and Its Applications.

[b15-j12dim] 15The approach via logarithms is generally the easiest.

[16] Gibbs JH, Di Marzio EA (1958). Chem J Phys.

[17] Di Marzio EA (1981). Annals NY Acad Sci.

[b18-j12dim] 18Provided we disallow ordering.

[b19-j12dim] 19Just what is or is not a species is a deep question. As far as the counting requirements of statistical mechanics are concerned molecules in different orientations can be considered to be distinct species.

[20] Nemirovski AM, Huston SE, Graham RL, Freed KF Packing Rods on d-dimensional Lattices: From Direct Enumeration to Series Expansion (Preprint).

[21] Flory PJ, Ronca G (1979). Mol Cryst Liquid Cryst.

[22] Li W, Freed KF, Nemirovski AM Packing Entropy of Hard, Extended Objects on a Lattice (Preprint).

[23] Mahanty J, Ninham BW (1976). Dispersion forces.

